# Effects of a 5-HT_1B_ Receptor Agonist on Locomotion and Reinstatement of Cocaine-Conditioned Place Preference after Abstinence from Repeated Injections in Mice

**DOI:** 10.3389/fnsys.2017.00073

**Published:** 2017-10-10

**Authors:** Taleen S. Der-Ghazarian, Tanessa Call, Samantha N. Scott, Kael Dai, Samuel J. Brunwasser, Sean N. Noudali, Nathan S. Pentkowski, Janet L. Neisewander

**Affiliations:** School of Life Sciences, Arizona State University, Tempe, AZ, United States

**Keywords:** serotonin, CP94253, sensitization, withdrawal, addiction, place conditioning

## Abstract

5-HT_1B_ receptors (5-HT_1B_Rs) modulate behavioral effects of cocaine. Here we examined the effects of the 5-HT_1B_R agonist 5-propoxy-3-(1,2,3,6-tetrahydro-4-pyridinyl)-1H-pyrrolo[3,2-b]pyridine (CP94253) on spontaneous and cocaine-induced locomotion and on cocaine-primed reinstatement of conditioned place preference (CPP) in male mice given daily repeated injections of either saline or cocaine (15 mg/kg, IP) for 20 days. In the locomotor activity experiment, testing occurred both 1 and 20 days after the final injection. In the CPP experiment, mice underwent conditioning procedures while receiving the last of their daily injections, which were given either during or ≥2 h after CPP procedures. The CPP procedural timeline consisted of baseline preference testing (days 12–13 of the chronic regimen), conditioning (days 14–19, 2 daily 30-min sessions separated by 5 h), CPP test (day 21), extinction (days 22–34; no injections), CPP extinction test (day 35), and reinstatement test (day 36). Mice that had not extinguished received additional extinction sessions prior to reinstatement testing on day 42. On test days, mice were pretreated with either saline or CP94253 (10 mg/kg, IP). Testing began 30 min later, immediately after mice were primed with either saline or cocaine (5 mg/kg for locomotion; 15 mg/kg for reinstatement). We found that CP94253 increased spontaneous locomotion in mice receiving repeated injections of either saline or cocaine when tested 1 day after the last injection, but had no effect on spontaneous locomotion after 20 days abstinence from repeated injections. Surprisingly, cocaine-induced locomotion was sensitized regardless of whether the mice had received repeated saline or cocaine. CP94253 attenuated expression of the sensitized locomotion after 20 days abstinence. A control experiment in noninjected, drug-naïve mice showed that CP94253 had no effect on spontaneous or cocaine-induced locomotion. Mice reinstated cocaine-CPP when given a cocaine prime, and CP94253 pretreatment attenuated cocaine reinstatement.The findings suggest that stress from repeated saline injections and/or co-housing with cocaine-injected mice may cross-sensitize with cocaine effects on locomotion and that CP94253 attenuates these effects, as well as reinstatement of cocaine-CPP. This study supports the idea that 5-HT_1B_R agonists may be useful anti-cocaine medications.

## Introduction

Serotonin plays a role in the reinforcing and incentive motivational effects of cocaine and cocaine-associated cues (Markou et al., 1993; Shaham et al., 2003). One mechanism involved in these effects is the action of serotonin at 5-HT_1B_ receptors (5-HT_1B_Rs; Clark and Neumaier, 2001; Filip et al., 2010; Miszkiel et al., 2011; Neisewander et al., 2014). Parsons et al. (1998) discovered that 5-HT_1B_R agonists shift the cocaine self-administration (SA) dose-effect function to the left and increase responding on a PR schedule of cocaine reinforcement, suggesting enhanced reinforcing value of cocaine. These 5-HT_1B_R agonist effects are reversed by a 5-HT_1B_R antagonist, demonstrating that they are 5-HT_1B_R-mediated. Furthermore, the agonists do not alter sucrose or food reinforcement or locomotion at doses that enhance the reinforcing value of cocaine (Parsons et al., 1998; Przegaliński et al., 2007; Pentkowski et al., 2009). Surprisingly, we found that both cue and cocaine-primed reinstatement of cocaine-seeking behaviors are attenuated by 5-HT_1B_R agonists (Acosta et al., 2005; Pentkowski et al., 2009). These seemingly paradoxical findings led us to discover that 5-HT_1B_Rs modulate cocaine-related behaviors in opposite directions depending on whether or not animals have undergone an abstinence period prior to testing (Pentkowski et al., 2014). Specifically, either the agonist 5-propoxy-3-(1,2,3,6-tetrahydro-4-pyridinyl)-1*H*-pyrrolo[3,2-*b*]pyridine (CP94253) or viral overexpression of 5-HT_1B_Rs tested during the maintenance of daily SA sessions *increased* the reinforcing value of cocaine, measured as a leftward shift of the cocaine SA dose-effect function on low ratio schedules of reinforcement and an increase in intake on a progressive ratio schedule (Pentkowski et al., 2012, 2014). In contrast, after a 21-day period of protracted abstinence, the agonist *attenuated* cocaine intake at the same low dose of cocaine (0.075 mg/kg, IV) for which CP94253 had enhanced intake prior to an abstinence period (Pentkowski et al., 2014) and attenuated intake on a progressive ratio schedule of cocaine reinforcement. These findings demonstrate opposite functional effects of 5-HT_1B_R agonists pre- vs. post-abstinence from cocaine SA.

5-HT_1B_Rs also modulate spontaneous locomotion and cocaine-induced locomotion under some circumstances. Several studies have found that 5-HT_1B_R agonists stimulate locomotor activity in drug-naïve rats (Oberlander et al., 1986, 1987; Macor et al., 1990; Koe et al., 1992; Geyer, 1996; Chaouloff et al., 1999), but have no effect on spontaneous locomotion in rats with a history of cocaine SA (Przegaliński et al., 2007; Pentkowski et al., 2009). 5-HT_1B_R agonist effects on spontaneous locomotion may be specific to rats since the drugs have no effect in drug-naïve mice (Bannai et al., 2007; Fish et al., 2008; Nasehi et al., 2017). However, in mice that had been stressed by repeated behavior testing, CP94253 increases locomotion (Tatarczyńska et al., 2004, 2005). Additionally, the 5-HT_1A/1B_R agonist RU24969 dose-dependently increases spontaneous locomotion in wild type mice, but not 5-HT_1B_R knockout mice (Saudou et al., 1994). CP94253, as well as another 5-HT_1B_R agonist CP93129, have been shown to potentiate cocaine-induced locomotion and cocaine sensitization in rats (Przegaliński et al., 2001, 2002, 2004; Filip et al., 2010). Collectively, these findings suggest that 5-HT_1B_R stimulation enhances locomotion in rodents given cocaine or with a history of stress.

One goal of the present study was to examine whether the abstinence-induced “switch” in 5-HT_1B_R functional modulation of cocaine-related behaviors observed in rats previously is also observed in mice. To this end, we investigated whether CP94253 produces opposing effects on spontaneous and cocaine-induced locomotion before and after an abstinence period in C57BL/6 male mice receiving daily injections of either saline or cocaine (15 mg/kg, IP) for 20 days. The second goal was to investigate whether the incentive motivational effects of a cocaine priming injection are attenuated by 5-HT_1B_R agonist treatment in mice that had undergone abstinence, similar to the decrease in cocaine-primed reinstatement of cocaine-seeking behavior observed previously in rats (Pentkowski et al., 2012, 2014). To this end, we investigated CP94253 effects on cocaine-primed reinstatement of extinguished cocaine-conditioned place preference (CPP).

## Materials and Methods

### Animals

Male C57BL/6 mice arrived at 5 weeks old from Jackson Laboratories (Sacramento, CA, USA) and were group housed 3–4/cage in a climate-controlled facility with a reversed 10 h light/14 h dark cycle (lights off at 6:00 AM). Mice were handled for 2 weeks. For the CPP experiment only, mice were transferred to single housing 1 day prior to the start of behavior testing. Food and water were provided *ad libitum* in the home cage. All behavioral testing occurred between 8 AM and 4 PM. Separate groups of experimentally naïve mice were used for each specific experiment. All husbandry and procedures adhered to the National Research Council (US) Committee for the Update of the Guide for the Care and Use of Laboratory Animals (2011), and all experimental procedures were reviewed and approved by the Institutional Animal Care and Use Committee at Arizona State University.

### Drugs

Cocaine hydrochloride (RTI International, Research Triangle Park, NC, USA) and CP94253 (Tocris Bioscience, Minneapolis, MN, USA) were dissolved in bacteriostatic saline. All drugs were injected at a volume of 10 ml/1 kg of body weight. The doses used had been previously reported to produce cocaine- (Tilley et al., 2007; Shuman et al., 2012; Rao et al., 2013) and CP94253-induced hyperlocomotion in mice injected 30 min before testing (Tatarczyńska et al., 2004, 2005; Bannai et al., 2007; Fish et al., 2008).

### Apparatus

Locomotor activity tests were conducted in Plexiglas chambers, each measuring 35 × 24 × 31 cm high. The chambers had corn cob bedding on an acrylic floor and alternating black and white stripes on the walls. CPP experiments were conducted in Plexiglas two-compartment apparatus with each end compartment measuring 35 × 24 × 31 cm high and with a removable partition separating them. One compartment had cedar bedding beneath a wire 1 × 1 cm grid floor and alternating black and white vertical stripes on the walls. The other compartment had pine bedding beneath a parallel bar floor (5 mm diameter) and alternating black and white horizontal stripes on the walls. In order to prevent the mice from escaping from the chambers, while maintaining the ability to record their behavior via an overhanging video camera, a rectangular tower measuring 70 × 24 × 74 cm high of clear Plexiglas was used as an extension of the apparatus. The testing room was dimly lit with two overhead lamps, each containing a 25 Watt light bulb. A camera (Panasonic WV-CP284, color CCTV, Suzhou, China) used to record testing sessions was mounted 101 cm above the center of each apparatus. A WinTV 350 personal video recorder (Hauppage, NJ, USA) captured live video encoded into MPEG streams. A modified version of TopScan Software (Clever Sys Incorporated, Reston, VA, USA) was used to track the animals’ movement. This program uses the orientation of an animal’s body parts (e.g., nose, head, center of body, forepaws, base of tail, etc.) to identify the animal’s location and specified behaviors.

### Experiment 1: Effects of CP94253 on Spontaneous and Cocaine-Induced Locomotion before and after Chronic Daily Injections of Cocaine or Saline

**Figure 1:**
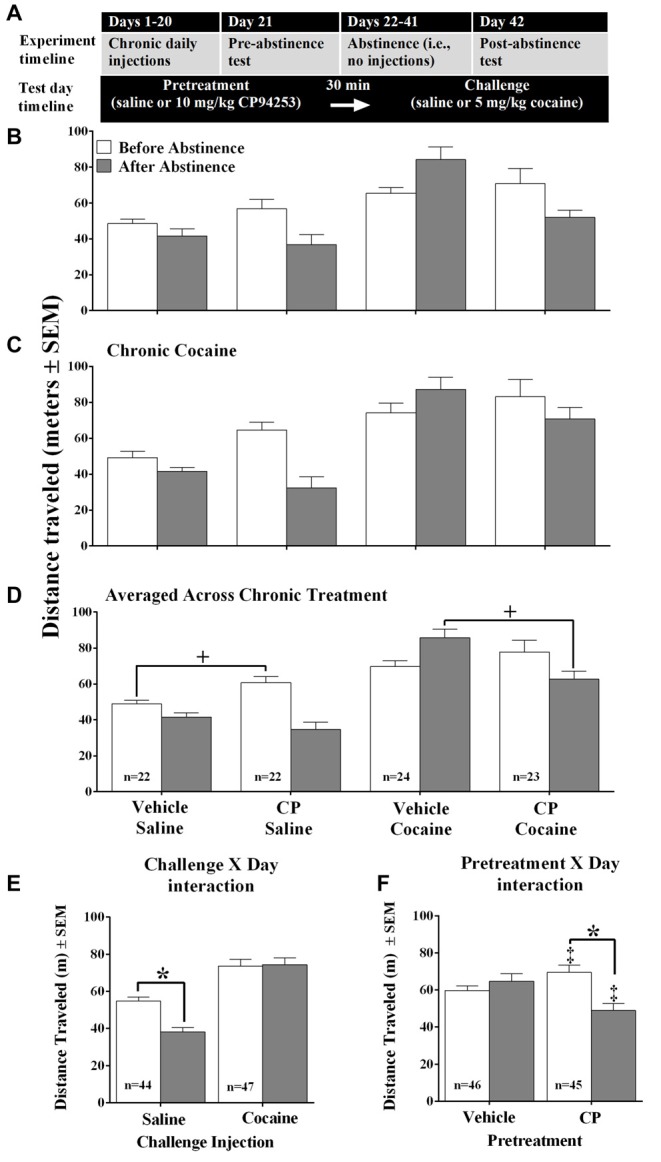
Timeline for Experiments 1–2 **(A)** and distance traveled (meters ± SEM) by mice that received either chronic daily injections of saline **(B)** or 10 mg/kg cocaine **(C)** and were tested both 24 h after the last of 20 injections (i.e., before abstinence, white bars) and 20 days after (i.e., after abstinence, gray bars), *n* = 8–11/group. Contrary to prediction, there was no effect of chronic treatment conditions nor interactions with cocaine challenge (0 or 5 mg/kg, IP) or 5-propoxy-3-(1,2,3,6-tetrahydro- 4-pyridinyl)-1H-pyrrolo[3,2-b]pyridine (CP94253) (0 or 10 mg/kg, IP), so further analyses were conducted averaged across the chronic treatment variable **(D)**. This analysis yielded a challenge injection by day interaction **(E)** and a pretreatment by day interaction **(F)**. Asterisk (*) represents a significant *post hoc* comparison, *p* < 0.05; Plus sign (+) represents a significant planned comparison, *p* < 0.05. Double plus (‡) represents a significant difference from respective vehicle condition, Bonferroni *t*-test *p* < 0.001.

The timeline for Experiment 1 is shown in Figure 1A. Adult, male C57BL/6 mice (*n* = 91) were housed four/cage, with two mice in each cage assigned to receive saline and two assigned to receive cocaine (15 mg/kg, IP) at the same time of day for 20 consecutive days. The mice were further assigned to receive two different pretreatments on the test days. The first pretreatment was either vehicle or CP94253 (10 mg/kg, IP) and the second pretreatment was either a saline or cocaine (5 mg/kg, IP) challenge injection. Thus the design of this experiment was a 2 (chronic saline or cocaine) × 2 (vehicle or CP94253 pretreatment) × 2 (saline or cocaine challenge) factorial with eight treatment groups (*n* = 8–11/group). Test day 1 took place on the day after the last chronic injection. After test 1, the mice underwent a 20-day period of no injections during which they remained in the colony room and their tails were marked twice per week to maintain identification. Test day 2 took place the day after the final abstinence (i.e., no injection) day. On both of the test days, mice were first placed into the test chamber for 1 h to allow for habituation. Immediately following this baseline period, mice were injected with either vehicle or CP94253 and were returned to their home cage for 30 min. Next, mice received the saline or cocaine challenge injection and were returned to the test chamber for an additional 60 min. We used a lower cocaine dose for the challenge (5 mg/kg) on test day than that used during the daily repeated administration (15 mg/kg). This was done in order to avoid potential ceiling effects for detecting sensitization of locomotion, a well-known effect of repeated cocaine administration (Ago et al., 2008; DiRocco et al., 2009; Luo et al., 2010; Thompson et al., 2010; Riday et al., 2012; Robison et al., 2013).

### Experiment 2: Effects of CP94253 on Spontaneous and Cocaine-Induced Locomotion in Mice without the Repeated Injection Regimen

In order to assess potential injection stress effects, we repeated Experiment 1 using identical procedures and timeline except that the 5 week old, male C57BL/6 mice (*n* = 47) did not receive any injections during the first 20 days of the experiment. Thus, the four mice/cage were simply handled twice a week to color-mark tails for identification purposes and were otherwise left undisturbed to minimize stress. The design was a 2 (vehicle or CP94253 pretreatment) × 2 (saline or cocaine challenge) factorial with four treatment groups (*n* = 11–12/group). Test day procedures were identical to Experiment 1.

### Experiment 3: Effects of CP94253 on Reinstatement of Extinguished Cocaine-CPP

The timeline for Experiment 3 is shown in Figure 3A. Adult, male C57BL/6 mice received daily injections of cocaine (15 mg/kg, IP) or saline for 11 days in order to keep the same number of cocaine injections prior to testing for effects of CP94253 in this experiment as that given in the previous experiments. Additionally, the mice were housed three/cage and all three mice/cage were assigned to the same chronic drug condition. On day 12 and 13 the mice were allowed free access to both sides of the CPP apparatus for 15 min to habituate them to the novel environments and to assess initial compartment preference. The average of the time spent in the least preferred compartment on days 12 and 13 was used as the baseline preference measure. On both days 12 and 13, mice received their chronic daily injection (saline or cocaine) in their home cage 2–3 h after the preference test. On days 14–19, the mice underwent two daily 30-min conditioning sessions separated by a 5-h period. During the morning session, mice were injected with saline and were placed into their initially preferred side and during the afternoon session mice were injected with cocaine (15 mg/kg, IP) or saline and were placed into their initially non-preferred side. On day 20, mice were not exposed to the apparatus, but did receive either saline or cocaine (15 mg/kg, IP) at the same time of day as all previous injections. On day 21, mice were tested for the expression of cocaine CPP for 15 min. Only 80% of the mice met the CPP expression criterion (spent >450 s in initially non-preferred compartment) and continued in the experiment. These mice next underwent extinction training on Days 22–34. During extinction, the mice received one 30-min exposure to one of the compartments each day, with the particular compartment alternating across the days. On day 35, mice were tested for 15 min to demonstrate that their CPP had extinguished. Mice that extinguished were tested for reinstatement of CPP the following day (day 36). On test day, mice received either saline or CP94253 (10 mg/kg, IP) 30 min prior to the test. Immediately before the test, the mice were primed with either saline or cocaine (15 mg/kg, IP). Mice that did not initially extinguish received four more days of extinction with two, 30-min sessions per day, one in each compartment. They again received a 15-min preference test to demonstrate that their CPP had extinguished. Mice that extinguished were tested for reinstatement of CPP the following day. Mice that failed to extinguish were removed from the study. The design of the study was a 2 (vehicle or CP94253 pretreatment) × 2 (saline or cocaine challenge) factorial with four treatment groups (*n* = 9–11/group). Additionally, a group of mice (*n* = 14) were treated chronically with saline, conditioned with saline during both daily sessions, extinction-trained, and given a saline prime prior to testing (i.e., saline control group).

**Figure 2:**
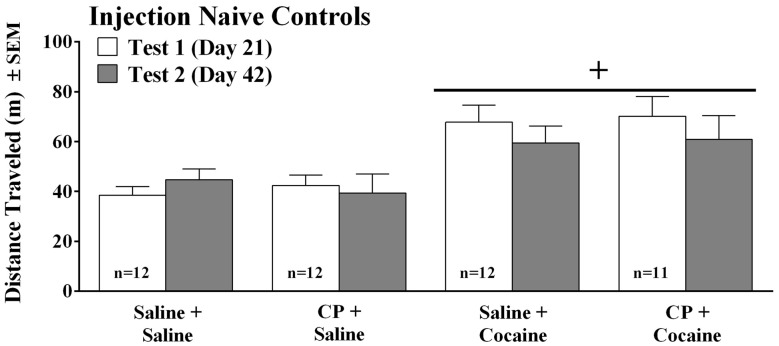
Distance traveled (meters ± SEM) by injection-naïve mice that were treated the same as mice in the previous experiment (see timeline on Figure 1) except that they were not given daily injections over the first 20 days of the experiment, but were instead left undisturbed in their home cages except for twice weekly tail marking for identification. On the test days, the mice received an injection of either saline or CP94253 (10 mg/kg, IP) and 30 min later received a saline or cocaine (5 mg/kg, IP) injection (*n* = 11–12/group). Plus sign (+) represents a significant difference from saline-challenged groups, *p* < 0.001.

**Figure 3:**
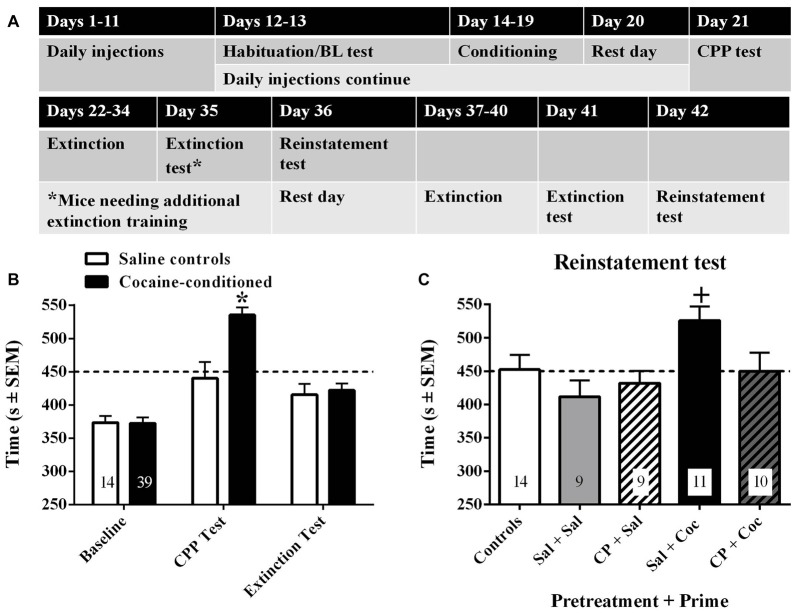
Timeline for Experiment 3 **(A)** and results of 15-min preference tests to assess baseline preference, cocaine-conditioned placepreference (CPP), and extinction of cocaine-CPP **(B)**. Subsequently, mice that had received repeated saline injections (white bars) or repeated cocaine injections (black bars) prior to and during conditioning were tested for reinstatement of CPP **(C)** following pretreatment with either saline or CP94253 (10 mg/kg, IP) and a priming injection of either saline (Sal) or cocaine (15 mg/kg, IP; Coc) 30 min later and immediately prior to the test (*n* = 9–11/group). Values are the time (s ± SEM) in the initially non-preferred compartment (i.e., cocaine-paired side for conditioned mice) and dashed line represents 50% of the total test time such that values above the line illustrate a preference switch. Asterisk (*) represents difference from saline group, Bonferroni *t*-test *p* < 0.001. Plus (+) represents difference from all other groups, Tukey test, *p* < 0.05.

### Statistics

Drug-induced changes in distance traveled (meters) were analyzed and graphed for the first 30 min of each testing session. Only the first 30 min of the testing sessions were analyzed because cocaine is rapidly metabolized in mice (Tilley et al., 2007; Rao et al., 2013) and the difference from baseline calculation controlled for individual differences in baseline activity. The changes in distance traveled measures were analyzed by mixed factor analysis of variances (ANOVAs) with the following between group variables: chronic treatment with cocaine or saline (Experiment 1 only); pretreatment with CP92453 or vehicle; challenge with cocaine or saline prior to test. The ANOVAs also included Test day as a within subjects repeated measure. Interactions were further analyzed by smaller ANOVAs and *t*-test with Bonferroni correction for multiple comparisons where appropriate. In addition, planned comparisons were conducted to test our hypothesis that CP94253 would enhance spontaneous locomotion and cocaine-induced locomotion pre-abstinence, but would have the opposite effect post-abstinence. Mice whose distance traveled score was more than ±2 standard deviation from the mean were deemed outliers and removed from all analysis. For CPP, time spent in the initially non-preferred side was analyzed by ANOVA with test days as a repeated measure. The test days included the baseline preference test, the CPP test (occurred after six daily pairings with cocaine), and the extinction test (occurred after 18–22 sessions of extinction). This analysis was a manipulation check to demonstrate that cocaine-conditioned rats exhibited CPP and extinction of CPP. To analyze cocaine-primed reinstatement of CPP, time spent in the initially non-preferred compartment of the apparatus (drug-paired compartment) was analyzed by a 2 (Pretreatment: CP94253 and vehicle) × 2 (Priming injection: Cocaine and saline) AVOVA. Interactions were analyzed by smaller ANOVAs and Tukey *post hoc* tests.

## Results

### Experiment 1: Effects of CP94253 on Spontaneous and Cocaine-Induced Locomotion before and after Chronic Daily Injections of Cocaine or Saline

We first tested the hypothesis that mice given chronic cocaine treatment would exhibit a “switch” in 5-HT_1B_R agonist effects from facilitation of cocaine-induced locomotion during the treatment phase to inhibition of cocaine-induced locomotion after a period of abstinence from chronic cocaine. Surprisingly, the chronic saline group behaved similarly to the chronic cocaine group (Figures 1B,C) and the analysis confirmed that there was no main effect nor interactions with chronic treatment (i.e., chronic saline vs. cocaine). Therefore, subsequent analyses were conducted with the data are averaged across chronic condition as shown in Figure 1D. This analysis revealed a main effect of Challenge, where the cocaine challenge increased locomotion compared to the saline challenge when averaged across pretreatment with Vehicle or CP94253 (*F*_(1,87)_ = 62.28, *p* < 0.001). However, there was also a Challenge by Day interaction (*F*_(1,87)_ = 15.47, *p* < 0.001) as shown in Figure 1E. Subsequent pairwise comparisons with Bonferroni correction indicated that cocaine-challenged mice showed no difference in locomotion across test days, whereas saline challenged mice showed a decrease in locomotion after abstinence compared to before abstinence (*t*_(43)_ = 5.8, *p* < 0.001). There was also a Pretreatment by Day interaction (*F*_(1,87)_ = 32.83, *p* < 0.001) as shown in Figure 1F. Subsequent pairwise comparisons indicated that mice pretreated with vehicle showed no difference in locomotion across test days, whereas mice pretreated with CP94253 showed less locomotion after abstinence compared to before abstinence (Bonferroni *t*-test, *t*_(44)_ = 5.8, *p* < 0.001). In addition to the ANOVAs, planned comparisons were conducted to test the hypothesis that CP94253 pretreatment would facilitate spontaneous and cocaine-induced locomotion before abstinence but inhibit these behaviors after abstinence. The results of these comparisons indicated that there was a significant increase in spontaneous locomotion after the CP94253 pretreatment compared to vehicle pretreatment in mice challenged with saline before abstinence from repeated injections (*t*_(42)_ = 3.0, *p* < 0.01, Figure 1D). In mice challenged with cocaine, there was no difference in cocaine-induced locomotion between vehicle- and CP94253-pretreated mice before abstinence, but the CP94253-pretreated mice showed less cocaine-induced locomotion than vehicle-pretreated mice after abstinence (*t*_(45)_ = 3.6, *p* < 0.05, Figure 1D).

### Experiment 2: CP94253 Has no Effect in Mice that Have Not Undergone a Repeated Injection Regimen

The finding that chronic cocaine vs. chronic saline treatment did not show differences in locomotion in the previous experiment was puzzling. We reasoned that stress experienced by the saline control group may have cross-sensitized the mice to cocaine such that both groups (i.e., chronic cocaine and chronic saline) showed sensitized responses to cocaine (Sorg, 1992). Indeed, the control mice experienced repeated injections and were housed with cocaine-treated mice, and both of these manipulations are chronic stressors in mice (Ryabinin et al., 1999; Hoplight et al., 2007). Another concern was that rather than CP94253 having opposite effects on cocaine-induced locomotion before and after abstinence from repeated injections, perhaps the agonist simply has opposite effects the first time it is administered compared to the second time it is administered. We examined these possibilities in this experiment. Naïve, non-injected mice arrived at the same age as in the previous experiment and were housed for 20 days during which they were handled twice weekly to color-mark tails for identification purposes and were otherwise left undisturbed. As expected, cocaine increased locomotion to a similar degree on the first (day 21) and second (day 42) test days as there was a main effect of Challenge (*F*_(1,43)_ = 15.15, *p* < 0.001), but no interactions with Pretreatment or Day. In contrast to the effects of CP94253 observed in the repeatedly injected saline controls (Figure 1B), CP94253 had no effects on locomotion in injection-naive mice (Figure 2). This finding suggests that the saline injections in mice from the previous experiment did indeed produce stress that affected spontaneous and cocaine-induced locomotor activity in a 5-HT_1B_R-sensitive manner.

### Experiment 3: CP94253 Prevents Cocaine-Primed Reinstatement of Extinguished Cocaine CPP

Approximately 40% of the mice preferred the side of the apparatus with horizontal stripes and ~60% preferred the side with vertical stripes, confirming the use of an unbiased apparatus. A repeated measures analysis across the baseline, CPP, and extinction tests showed a significant day by conditioning treatment interaction (*F*_(2,106)_ = 13.23, *p* < 0.001; Figure 3B). Subsequent analyses comparing saline to cocaine conditioned groups on each test day showed a group difference on the CPP test day but no difference during baseline or extinction (Bonferroni *t*-test *t*_(51)_ = 3.98, *p* < 0.001). These results indicate that cocaine conditioning produced CPP that was abolished by extinction training. In the cocaine conditioned groups, a 2 × 2 ANOVA of time spent in the drug-paired side during the reinstatement test revealed a significant Pre-treatment × Priming injection interaction (*F*_(1,35)_ = 4.26, *p* < 0.05; Figure 3C). Subsequent *post hoc* analyses indicated that the cocaine-primed, saline-pretreated group showed significantly greater CPP than all other groups (Tukey tests, *p* < 0.05). In addition, comparisons of each group to its extinction baseline indicated that only the cocaine-primed group showed a significant increase in time spent in the drug-paired side relative to extinction baseline (*t*_(10)_ = 4.1, *p* < 0.005). Finally, the cocaine-primed, saline-pretreated group also showed a significantly greater amount of time spent in the drug-paired side relative to the saline controls (*t*_(23)_ = 2.4, *p* < 0.05). These results suggest that CP94253 attenuated cocaine-primed reinstatement of cocaine CPP.

## Discussion

This study yielded partial support for our hypothesis that mice would show a similar abstinence-dependent change in 5-HT_1B_R modulation of cocaine effects as observed previously in rats (Pentkowski et al., 2009, 2012, 2014). We predicted that the 5-HT_1B_R agonist CP94253 would facilitate cocaine-induced locomotion in mice given repeated daily injections of cocaine, but would inhibit this behavior after a 20-day period of abstinence, similar to the “switch” in 5-HT_1B_R agonist effects observed in rats before and after abstinence from cocaine SA. Surprisingly, we found that CP94253 effects on locomotion were the same regardless of whether or not the mice received repeated injections of saline or cocaine (Figures 1B,C). We then conducted further analyses without the chronic treatment as a factor (Figure 1D). We found that acute administration of CP94253 initially increased spontaneous locomotion in mice tested on the 21st day of their chronic injections as predicted; however, the agonist did not alter spontaneous locomotion after a 21-day abstinence phase. Furthermore, the effects of the agonist on cocaine-induced locomotion only partially supported our predictions because CP94253 failed to alter this behavior initially, but did reverse the cocaine-sensitized hyperlocomotion observed after 20 days abstinence from daily repeated injections. Overall, the results are generally consistent with previous findings in rats of a facilitatory effect on cocaine-induced behavior prior to abstinence and an inhibitory effect after a prolonged period of abstinence.

We had expected that the chronic repeated cocaine injections would sensitize mice to the cocaine challenge given on the first test day and that this effect would be evident as greater locomotor activity in the chronic cocaine-injected group relative to the chronic saline-injected control group. Because there was no difference between these groups, we speculated that our chronic repeated saline injections may have stressed the mice in the experiment resulting in stress-induced cross-sensitization. Previous research has demonstrated cross-sensitization between repeated stress and repeated cocaine injections in both rats and mice (Sorg, 1992; Prasad et al., 1995; Kikusui et al., 2005; Maeda et al., 2006; Boyson et al., 2014), and repeated injections are stressful in both mice and rats (Ryabinin et al., 1999; Ferguson et al., 2009). Another possible stressor was that the control mice were cohoused with the cocaine-treated mice, which may have resulted in chronic social stress. Although we did not notice overt signs of stress such as aggression, Hoplight et al. (2007) have previously shown that saline-injected rats pair housed with cocaine-injected rats have altered 5HT_1B_R profiles similar to that of cocaine treated rats, but not those housed with saline treated rats. To test this stress cross-sensitization hypothesis, we examined spontaneous and cocaine-induced locomotion in mice that were group housed and left undisturbed for 20 days except for tail-marking twice/week. In these control mice, the second cocaine challenge failed to sensitize locomotion in contrast to the sensitized locomotion observed in mice that were co-housed with cocaine-injected mice and given chronic saline injections. Furthermore, CP94253 failed to alter either spontaneous or cocaine-induced locomotion on either test day in the noninjected control mice. It is important to note that these control mice were tested on two separate occasions after receiving CP94253 pretreatment, mitigating the idea that CP94253 may simply produce different effects the first vs. second time it is given. The different pattern of behavior across the chronic saline-injected and noninjected mice, coupled with the similar pattern of behavior in the chronic cocaine-injected and chronic saline-injected mice, support the interpretation that stress from repeated injection and living with cocaine-injected mice cross-sensitized the mice to cocaine. CP94253 reversed expression of the sensitized locomotion after a period of abstinence. Although the neural mechanisms underlying the stress cross-sensitization effects will require further investigation, one likely pathway contributing to these effects is the 5-HT_1B_R-expressing medium spiny neurons projecting from nucleus accumbens (NAc) shell to the VTA. Previous research has shown that 5-HT_1B_R located on GABAergic projection neurons from the NAc shell to the VTA may mediate stress cross-sensitization with psychostimulant drugs (Furay et al., 2011; Miczek et al., 2011; Nair et al., 2013).

Although we had predicted that CP94253 would attenuate cocaine-sensitized locomotion after a period of abstinence, a previous study by Przegaliński et al. (2001) showed that while CP94253 dose-dependently enhances hyperlocomotion produced by acute amphetamine administration in mice, it does not affect amphetamine sensitization. The present findings seem discrepant with those of Pentkowski et al. (2009, 2012) however, we suggest that CP94253 may differentially alter locomotion induced by cocaine vs. amphetamines based on recent work from our laboratory demonstrating a different pattern of changes in cocaine vs. methamphetamine SA. Unlike the enhancement of cocaine SA prior to abstinence, CP94253 reduces methamphetamine SA both before and after abstinence (Garcia et al., 2017).

As we had predicted, CP94253 attenuated the cocaine-primed reinstatement of extinguished cocaine-CPP in mice that had a history of chronic cocaine administration followed by protracted abstinence prior to testing. Neither CP94253 pretreatment alone nor a saline prime prior to reinstatement testing altered preference. These control data suggest that reinstatement was specific to cocaine priming and that CP94253 specifically reversed the cocaine priming effect rather than nonspecifically altering preference. The findings are consistent with previous research suggesting that 5-HT_1B_R agonists attenuate incentive motivational effects of cocaine priming injections in the operant extinction/reinstatement model (Przegaliński et al., 2002, 2007; Pentkowski et al., 2014). Collectively, the studies suggest that 5-HT_1B_Rs modulate the incentive motivational effects of a cocaine prime in both rats and mice (Parsons et al., 1998; Fletcher et al., 2002; Pentkowski et al., 2012, 2014).

Demonstrating effects of 5-HT_1B_R agonists on psychostimulant-induced and conditioned behaviors in mice is important because transgenic mice are a valuable tool for investigating the neural mechanisms of these behaviors. A leading hypothesis for the effects of the agonists on cocaine-induced behaviors suggests that 5-HT_1B_Rs inhibit either GABAergic interneurons in the VTA or GABAergic medium spiny neurons projecting from the NAc to VTA, and this action disinhibits DA neurons (Parsons et al., 1999; Yan and Yan, 2001; Neumaier et al., 2002; O’Dell and Parsons, 2004; Barot et al., 2007; Hoplight et al., 2007). For instance, a microdialysis study suggests that stimulating 5-HT_1B_Rs in the VTA inhibits GABA release from the neurons that tonically inhibit mesolimbic DA neurons. This leads to disinhibition of the mesolimbic DA neurons, increasing dopaminergic transmission in the NAc (O’Dell and Parsons, 2004). Because viral-mediated overexpression of 5-HT_1B_Rs in this pathway attenuates cocaine intake after abstinence (Pentkowski et al., 2012), it is likely that cocaine abstinence causes adaptations within the 5-HT_1B_R→GABAR→DA circuit in the VTA, which may underlie the inhibitory effects of 5-HT_1B_R agonists on cocaine-induced behaviors that are observed following protracted abstinence. Transgenic mice may be useful in elucidating the neural circuitry involved in 5-HT_1B_R agonists effects on cocaine-induced behavior.

In conclusion, this study demonstrates that a 5-HT_1B_R agonist reverses expression of cocaine sensitization and blocks cocaine-primed reinstatement of cocaine-CPP in mice. These findings offer further support for the idea that serotonin inhibits incentive motivational effects of cocaine through an action at 5-HT_1B_Rs. Furthermore, this research suggests that 5-HT_1B_Rs may be a useful target for developing medications for cocaine use disorders and that mice are a useful model for screening the potential anti-cocaine therapeutic effects of 5-HT_1B_R agonists, as well as for investigating the neural mechanisms involved in 5-HT_1B_R-mediated inhibition of the incentive motivational effects of cocaine.

## Author Contributions

All authors had full access to all the data in the study and take responsibility for the integrity of the data and the accuracy of the data analysis. JLN, TSD-G and NSP: study concept and design. TSD-G, TC, SNS, KD, SJB and SNN: acquisition of data. TSD-G, JLN and SNP: analysis and interpretation of data. TSD-G and JLN: drafting of the manuscript. JLN and TSD-G: critical revision of the manuscript for important intellectual content. TSD-G and JLN: statistical analysis. JLN: obtained funding. TSD-G and JLN: study supervision.

## Conflict of Interest Statement

The authors declare that the research was conducted in the absence of any commercial or financial relationships that could be construed as a potential conflict of interest. The handling Editor declared a shared affiliation, though no other collaboration, with the authors and states that the process nevertheless met the standards of a fair and objective review.
