# Addendum: Effects of a 5-HT_1B_ Receptor Agonist on Locomotion and Reinstatement of Cocaine-Conditioned Place Preference after Abstinence from Repeated Injections in Mice

**DOI:** 10.3389/fnsys.2018.00048

**Published:** 2018-11-07

**Authors:** Taleen S. Der-Ghazarian, Tanessa Call, Samantha N. Scott, Kael Dai, Samuel J. Brunwasser, Sean N. Noudali, Nathan S. Pentkowski, Janet L. Neisewander

**Affiliations:** School of Life Sciences, Arizona State University, Tempe, AZ, United States

**Keywords:** serotonin, CP94253, sensitization, withdrawal, addiction, place conditioning

## Discrepancy in animal protocol

The authors have identified a discrepancy between the 15 mg/kg, IP cocaine priming dose used in this study, and the IACUC approved protocol dose of 10 mg/kg, IP. The dose of 15 mg/kg, IP cocaine used for the repeated drug injections in this study is correct and approved in the same IACUC protocol and caused no pain or discomfort to mice.

### Conflict of interest statement

The authors declare that the research was conducted in the absence of any commercial or financial relationships that could be construed as a potential conflict of interest. The handling Editor declared a shared affiliation, though no other collaboration, with the authors and states that the process nevertheless met the standards of a fair and objective review.

